# In vitro

**DOI:** 10.1186/cc12298

**Published:** 2013-03-19

**Authors:** D Winstedt, O Thomas, US Schött

**Affiliations:** 1 Lund University, Lund, Sweden

## Introduction

Rotational thromboelastography (ROTEM) can detect dilutive and hypothermic effects on coagulation and evaluate corrective treatments. The aim of this *in vitro *study was to study whether fibrinogen concentrate alone or combined with factor XIII could reverse colloid-induced and crystalloid-induced coagulopathies in the presence and absence of hypothermia.

## Methods

Citrated venous blood from 10 healthy volunteers was diluted by 33% using 130/0.42 hydroxyethyl starch or Ringer's acetate. ROTEM was used to evaluate the effect of addition of either fibrinogen concentrate corresponding to 4 g/70 kg, or this fibrinogen dose combined with factor XIII equivalent to 20 IU/kg. Blood was analyzed at 33 or 37°C with ROTEM ExTEM and FibTEM reagents.

## Results

A significant dilutive response was shown in both groups: hypocoagulation was greater in the starch group. Hypothermia lengthened the following: ExTEM clotting time (CT), clot formation time and α angle; FibTEM maximal clot formation (MCF). Irrespective of temperature, fibrinogen overcorrected Ringer's acetate's effects on all ROTEM parameters and partially reversed starch's effects on ExTEM CT and FibTEM MCF. FibTEM demonstrated that factor XIII provided an additional procoagulative effect in the Ringer's acetate group at both temperatures but not the starch group. The only ExTEM parameter to be improved by addition of factor XIII was MCF at 33°C.

## Conclusion

ROTEM shows that fibrinogen concentrate can reverse dilutive coagulation defects induced by colloid and crystalloid at both 33 and 37°C. Some additional reversal was provided by factor XIII: higher doses of both fibrinogen and factor XIII may counteract starch's effects on clot structure.

